# Dysregulation of adipokines in the serum of breast cancer patients – role in pathogenesis and potential clinical application (preliminary studies)

**DOI:** 10.3389/fimmu.2026.1724900

**Published:** 2026-03-31

**Authors:** Maria-Laura Morawiec, Dominika Wendlocha, Jacek Kabut, Aleksandra Mielczarek-Palacz

**Affiliations:** 1 Department of Immunology and Serology, Faculty of Pharmaceutical Sciences in Sosnowiec, Medical University of Silesia in Katowice, Katowice, Poland; 2 Department of Oncology and Radiotherapy, Medical University of Silesia, Katowice, Poland

**Keywords:** adipokines, adipsin, breast cancer, follistatin-like 1 (FSTL1), inflammation, isthmin, nesfatin, progranulin

## Abstract

**Introduction:**

The pathogenesis and diagnosis of breast cancer remain the subject of intensive research, as many aspects of these processes still require further elucidation. The dominant component of breast tissue stroma, adipose tissue, is composed of adipocytes, which secrete various cytokines, including adipokines, whose role in breast cancer is not fully understood.

**Methods:**

In this study, selected new adipokines were measured for the first time, including: progranulin (PGRN), follistatin-like protein 1 (FSTL1), asprosin (Asp), meteorin (METRN), adipsin (CFD), nesfatin (NES1), neuregulin 4 (NRG4), and isthmin (ISM1) in the serum of breast cancer patients, divided according to the molecular subtype and grade of the tumor. For the first time, the relationship between the adipokines studied and numerous standard laboratory parameters was also analyzed.

**Results:**

The study revealed a statistically significant difference in serum concentrations of adipsin and isthmin in breast cancer patients compared to the reference group, which may indicate systemic immune and inflammatory response disorders involving the studied adipokines.

**Discussion:**

The correlations between the studied adipokines in the course of breast cancer indicate their multifaceted effects on the pathogenesis of breast cancer, which requires further detailed research.

## Graphical abstract

**Figure:**
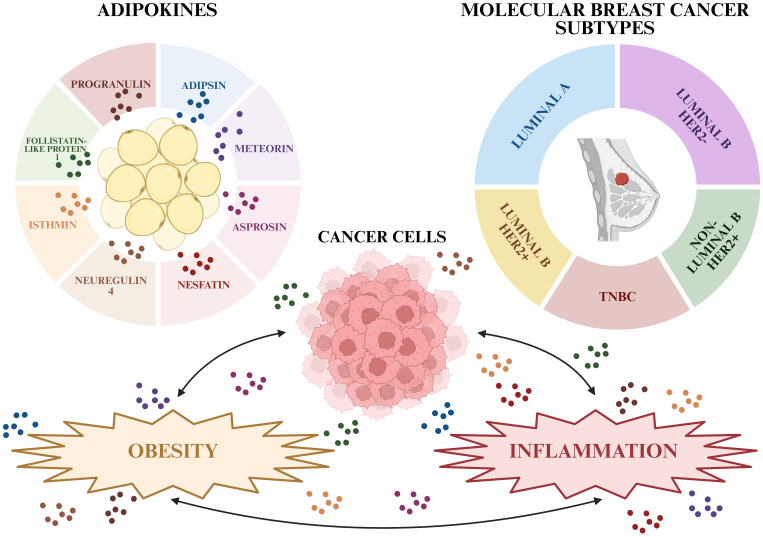
Created in https://BioRender.com.

## 1 Introduction

### 1.1 Breast cancer

Breast cancer (BC) was the second most commonly diagnosed cancer in women worldwide in 2022, accounting for 11.6% of all (1, 2). Breast cancer remains the most common cancer in women worldwide in terms of incidence (1). Not only is the incidence of cancer alarming, but also its high mortality rate, which is influenced by late stage diagnosis, distant metastases including bone marrow carcinomatosis, systemic relapses and treatment failures (3, 4). The pathogenesis of breast cancer involves genetic and environmental factors, the location of the tumor, and the presence of specific cells, including immune cells (5). The interactions between these cells in the developing tumor microenvironment (TME) also influence the etiology of the disease (5). Breast cancer can be divided into molecular subtypes, determined by: estrogen receptor (ER) expression, progesterone receptor (PR) expression, human epidermal growth factor receptor 2 (HER2) expression, and Ki-67 proliferation index expression, which are associated with the choice of treatment (6, 7). The classification recognizes at least five subtypes, including: triple-negative breast cancer (TNBC), luminal A (LA), luminal B (LB), HER-2-enriched (non-luminal), and normal breast-like cancer (6, 7). Based on histological type, breast cancer can be divided into: invasive ductal carcinoma (IDC), ductal carcinoma *in situ* (DCIS), invasive lobular carcinoma (ILC), inflammatory carcinoma, and other rare types (3). Diagnostics primarily involves imaging tests, such as mammography, one of the basic screening tests for breast cancer, magnetic resonance imaging (MRI), ultrasound (USG), histopathological tests and laboratory tests, including genetic testing (8). There are many risk factors that may increase the likelihood of developing breast cancer, including gender, age, family history, genetic mutations, and an unhealthy lifestyle (8). These risk factors for the development or recurrence of breast cancer also include being overweight and obese (9, 10). Obesity is inversely correlated with the risk of premenopausal breast cancer (10). However, compared to non-obese women with breast cancer, obese women with breast cancer have lower disease-free survival and overall survival rates and experience more complications related to surgery, radiotherapy, and chemotherapy (9, 11). Cholesterol is considered a risk factor for breast cancer, and high cholesterol levels have been linked to breast cancer metastasis (12).

Anatomically, breast tissue consists of adipose tissue, composed mainly of adipocytes, which form the breast stroma (13). Adipose tissue is a source of energy storage, but it is also an endocrine organ, secreting various cytokines, chemokines, and hormonal factors, i.e., adipokines (14). In adipose tissue, adipokines are involved in adipogenesis, immune cell migration, and adipocyte metabolism (13). In breast cancer, however, adipocytes stimulate cell growth, proliferation, and migration, and contribute to treatment resistance (15). Obesity, characterized by excessive accumulation of mature adipocytes, promotes the development of many cancers, including postmenopausal breast cancer, and is an independent risk factor for cancer progression and an indicator of poor clinical outcomes. It has also been associated with resistance to chemotherapy and radiotherapy (15–17). Obesity is also associated with a 35–40% increased risk of breast cancer recurrence and death (18). Obese patients have larger and more aggressive tumors, and more frequent lymph node metastases (18). Data demonstrating an association between obesity and breast cancer molecular subtypes are inconsistent (18). Potential mechanisms linking obesity to high cancer risk and mortality include obesity-related insulin resistance, hyperinsulinemia, hyperglycemia, glucose intolerance, oxidative stress, inflammation, and/or adipocytokine production (19). Adipose tissue inflammation, which occurs in most obese individuals, is closely associated with a poorer prognosis in breast cancer patients (20).

Adipokines, secreted by adipocytes, are the link between metabolism and the immune system. Their dysregulation, which occurs, for example, in obesity, contributes, among other things, to chronic inflammation (14). Adipocytes, especially visceral adipocytes in obese individuals, exhibit both local and systemic pro-inflammatory effects, characterized by abnormal production of pro-inflammatory factors, leading to changes in key signaling mediators, infiltration and activation of immune cells, stromal desmoplasia, and vasculitis, which promotes carcinogenesis and the formation of an appropriate TME, that supports tumor growth and progression (15, 16, 21). Cancer-associated adipocytes (CAAs) are a major component of the breast tumor TME and also contribute to tumor development (13, 17, 22, 23). Adipocytes enhance the mesenchymal phenotype, migration, and invasion of breast cancer cells through the C-X-C motif chemokine ligand 3 (CXCL3) derived from adipocytes and further activation of the focal adhesion kinase (FAK) pathway in breast cancer cells (23). Mature adipocytes may participate in mechanisms regulating breast cancer tumor growth through their effect promoting the growth of ER-positive cancer cells (24). The adipokines secreted by adipocytes and/or CAA and their interactions with breast cancer cells may contribute to the pathogenesis of the disease (13, 17, 22, 23). Research suggests that most adipokines promote cancer cell progression and invasion by enhancing cell proliferation and migration, inflammation, and anti-apoptotic pathways (19). In breast cancer, adipokines act in an endocrine or paracrine manner when secreted by non-breast and breast adipose tissue, respectively, and in an autocrine manner when secreted by cancer cells (13). The interaction between epithelial cells and adipocytes is characteristic of both normal breast tissue and breast cancer. In breast cancer, the interaction between cancer cells and adipocytes occurs during tumor initiation, progression, invasion, and metastasis (13).

The adipokines examined in this study include progranulin, follistatin-like protein 1, asprosin, meteorin, adipsin, nesfatin, neuregulin 4, and isthmin. Additional information on selected adipokines can be found in the **Supplementary Materials** section.

Adipokines may serve as biomarkers for breast cancer because they have been linked to inflammation, angiogenesis, insulin sensitivity, and mechanisms of innate and adaptive immunity (25). **Table 1** summarizes information on selected adipokines studied in breast cancer.

**Table 1:** Selected adipokines in breast cancer.

Adipokine	Role in breast cancer	Ref.
Progranulin	• induces secretion of inflammation-related cytokines such as interleukin (IL)-6 and -8 in a sortilin-dependent manner, IL-6 increases the population of breast cancer stem cells	(26)
	• promotes M2 macrophage polarization (pro-tumor), and programmed death-ligand 1 (PD-L1) expression by activating the STAT3 signaling pathway and through the interaction of programmed death receptor 1 (PD-1) with PD-L1, thereby promoting tumor immune escape	(27)
	• acts as an activator of cancer stem cells, highly secreted in ERα-negative BC, as well as in ERα-positive BC under hypoxic adaptation conditions	(28)
	• PGRN exposure causes dedifferentiation and increased proliferation of cancer stem cells	(28)
	• subcutaneous injections of PGRN or its active domain (GRN A) induce lung metastasis in BC xenograft models	(28)
	• stimulation of tumor cell proliferation, mediating their survival and conferring tamoxifen resistance; it also promotes metastasis and angiogenesis by stimulating vascular endothelial growth factor (VEGF)	(29)
	• high PGRN expression was associated with higher angiogenesis in BC, as reflected by increased VEGF expression and higher microvessel density	(30)
	• mediator of estrogen-induced proliferation in BC cells • associated with clinicopathological features of BC such as lymph node metastasis and tumor size • potential link to chemoresistance • independent prognostic factor	(3)
	• in PGRN−/− mice, BC metastasis to the lungs was inhibited PGRN−/− tumor-associated macrophages (TAMs) inhibited the invasion, migration, and EMT of BC cells through their exosomes • increased expression of miR-5100 in exosomes derived from PGRN−/− TAMs may regulate CXCL12 expression, inhibiting the CXCL12/CXCR4 axis and inhibiting invasion, migration, and epithelial-mesenchymal transition (EMT) of BC cells	(31)
	• drives the propagation of BC stem cells *in vitro* and increases metastasis formation in an *in vivo* breast cancer xenograft model • high expression of PGRN and sortilin was significantly associated with BC mortality, tumor size, high tumor grade, and lymph node metastasis • co-expression of PGRN and sortilin was not associated with tamoxifen resistance	(32)
	• interacts with DYRK1A, and their overexpression promotes TNBC cell proliferation and migration via miR-1246	(33)
	• stimulation of PGRN by lysophosphatidic acid (LPA) led to a significant increase in cell invasion in two of the three BC cell lines studied • incubation with anti-PGRN antibody and/or ERK (extracellular regulated kinase) pathway inhibitor inhibited the invasive capacity of BC cells	(34)
	• overexpression in BC cells resulted in the formation of cells capable of proliferating without estrogen and resistant to tamoxifen	(35)
	• tumor-derived PGRN was a key factor contributing to the exclusion of CD8+ T cells and resistance to anti-PD-1 treatment • tumor-derived PGRN promoted the polarization of TAM to M2 macrophages, and subsequently, PGRN-treated TAM inhibited the proliferation and activation of CD8+ T through ICAM-1 (intercellular cell adhesion molecule-1)-dependent interaction with CD8+ T cells in the TME • PGRN reduction alleviated the exclusion of CD8+ T cells in BC tissue and enhanced the response to anti-PD-1 treatment	(36)
	• induces cell proliferation and confers resistance to letrozole in a time- and dose-dependent manner • BC cells naturally resistant to letrozole showed a 10-fold increase in PGRN expression compared to letrozole-sensitive cells • does not affect aromatase enzymatic activity • blocks letrozole-induced downregulation of Bcl-2 • siRNA silencing reduces cell viability and restores sensitivity to letrozole	(37)
	• overexpression enabled BC cells proliferation in the absence of estrogen and in the presence of tamoxifen • blocked tamoxifen-induced apoptosis by preventing downregulation of Bcl-2 and cleavage of poly(ADP-ribose) polymerase • cells overexpressing PGRN showed higher levels of angiogenic factors: VEGF and angiopoietin-1	(38)
	• expression in the tumor at the time of diagnosis in patients with early-stage BC may provide additional information on survival beyond that obtained using the Nottingham Prognostic Index (NPI) alone, which takes into account lymph node status, tumor size, and histological differentiation	(39)
	• tissue expression in tumor biopsies is a prognostic factor for early recurrence	(40)
	• prognostic biomarker, predictive of recurrence risk and increased mortality in patients with ER+ IDC BC without metastases	(41)
	• stimulates HER2 phosphorylation and proliferation • confers resistance to trastuzumab in HER2-expressing cells • stimulates C-Myc phosphorylation and regulates C-MYC levels in HER2-expressing cells • stimulates C-SRC phosphorylation, a known upstream regulator of C-MYC	(42)
	• inhibits tamoxifen-induced apoptosis	(43)
FSTL1	• cancer metastases to the lungs significantly increased in mice with FSTL1 deficiency • may act as a cellular or molecular agonist or antagonist of TAM receptors in immune checkpoint therapy for BC	(44)
	• participates in bone metastasis – mediates tumor cell invasion and bone tropism and increases the population of bone marrow-derived pluripotent CD45−ALCAM+ mesenchymal stem cells	(45)
	• FSTL1 deficiency in medullary thymus epithelial cells (mTECs) impairs T cell development, promoting the metastasis of TNBC to the lungs	(46)
	• participates in the miR-137/FSTL1/integrin β3/Wnt/β-catenin signaling axis in BC cells, which regulates stem cell capacity and chemoresistance	(47)
	• has a minor effect on the proliferation of BC cells and vascular endothelial cells	(48)
Asprosin	• potential diagnostic marker in breast cancer with good differentiation ability between BC and healthy patients	(49)
METRN	• does not affect the destructive effect of doxorubicin on BC cells and in mice with BC tumors	(50)
Adipsin	• CFD and its effector, hepatocyte growth factor (HGF), promote tumor invasion in an autocrine manner, enhance the interaction of adipocytes with BC stem cells, and these interactions promote tumor development and progression	(17)
	• enhances the proliferation and properties of cancer stem cells (CSCs) in human patient-derived xenografts (PDX) derived from BC patients • inhibition of adipsin-dependent signaling by a specific inhibitor or silencing of adipsin expression in ADSC (adipose-derived stem cells) reduced the expression of CSC markers in BC cells	(51)
Nesfatin	• high expression is associated with poor BC prognosis • NUCB2 NUCB2/nesfatin-1 promoted metastasis *in vitro* and *in vivo*, while nesfatin-1 prevented cell metastasis disorders caused by NUCB2 depletion • NUCB2/Nesfatin-1 increased cholesterol synthesis via the mammalian target of rapamycin complex 1 (mTORC1) signaling pathway, contributing to BC migration and metastasis • NES1 antibody treatment hindered migration and invasion in BC cell lines • restores BC stemness in NUCB2-knockout cell lines • NUCB2 increases cell proliferation, migration, and invasion of BC cells • NUCB2 is stimulated by estrogens and plays an important role in the BC metastasis process	(52)
	• NUCB2 status is a prognostic factor in BC	(53)
Neuregulin 4	• enhances the antiproliferative effect of anti-ERBB2 neutralizing antibodies, i.e., trastuzumab and pertuzumab • specifically activates ErbB4, whose higher levels correlate with longer recurrence-free survival in patients with HER2-positive and luminal A BC	(54)
Isthmin	• in ductal breast carcinomas, decreased ISM-1 levels could promote tumor development possibly through blocking angiogenesis inhibition and promotion of apoptosis	(25)

## 2 Materials and methods

### 2.1 Study population and reference population

The study group consisted of 64 patients diagnosed with invasive breast cancer, and the control group consisted of patients with benign breast lesions. All patients were hospitalized at the Diagnostic Center of the District Hospital in Rybnik. The benign group consisted of 20 patients who had been diagnosed with lesions such as fibroadenoma, fibrosclerosis, apocrine metaplasia, intraductal papilloma, and simple cyst. Patients in both the malignant and benign lesion groups were diagnosed based on the presence of a solid breast tumor detected via imaging tests (breast ultrasound, mammography). At the first visit, medical history was obtained regarding major complaints, chronic diseases (including neoplastic and autoimmune diseases), medications used, and obstetric history. The inclusion criterion for both groups was the presence of a solid breast lesion requiring histopathological verification. Patients with autoimmune diseases or previous oncological treatment were excluded from the study (exclusion criteria). For all patients, laboratory tests including, an ultrasound-guided core needle biopsy of the breast tumor, and a fine-needle biopsy of the axillary lymph nodes (if metastases were suspected) were performed. All benign lesions were histopathologically confirmed. All patients in the malignant group underwent imaging studies to assess the presence of distant metastases. The histopathological examination included information on the histological type, malignancy grade (G1, G2, G3). Based on the clinical data, the stage of neoplastic disease was assessed according to the TNM classification. Molecular features included in the histopathological protocol allowed the patients to be classified into one of the following breast cancer types: luminal A, luminal B HER2-negative, luminal B HER2-positive, non-luminal HER2-positive, and TNBC.

Characteristics of the reference group and the study group by molecular subtype of breast cancer are presented in **Table 2**.

**Table 2:** Characterization of the study and the reference group.

Parameters		Reference group		Total study group	Molecular breast cancer subtype					*p* value *
					Luminal A	Luminal B HER2+	Non-luminal B HER2+	Luminal B HER2-	TNBC	
N (%)		16 (20%)		64 (80%)	23 (36%)	9 (14%)	2 (4%)	23 (36%)	7 (10%)	–
Age		44.0 (39.0-48.5)		64.0 (51.2-71.0)	60.0 (50.0-70.0)	64.0 (61.0-70.0)	52.0 (48.0-56.0)	65.0 (55.0-72.0)	71.0 (64.0-73.0)	**<0.001**
Weight (kg)		75.0 (66.0- 82.0)		74.3 ± 16.4	71.0 (62.0- 76.0)	79.5 (62.0- 84.0)	–	77,67 ± 21.4	68.5 (61.0- 73.5)	>0.05
Malignancy degree	**G1**		–	9 (14%)	5 (8%)	0 (0%)	0 (0%)	4 (6%)	0 (0%)	–
	G2		–	41 (63%)	18 (28%)	6 (9%)	1 (2%)	12 (18%)	4 (6%)	–
	G3		–	14 (23%)	0 (0%)	3 (5%)	1 (2%)	7 (11%)	3 (5%)	–
WBC (K/ul)		6.10 (5.50-6.50)		6.93 ± 1,94	6.85 ± 2.07	6.00 (4.90-6.30)	9.30 (7.50-11.10)	6.40 (5.6-8.2)	7.60 (7.3-9.6)	>0.05
HGB (g/dl)		14.0 (12.7-14.5)		13.75 ± 1.62	13.7 ± 1.4	13.1 (12.7-13.9)	14.4 (14.0-14.8)	13.9 ± 2.2	13.4 (13.3-13.9)	>0.05
PLT (K/ul)		273 (209-331)		254 (221-308)	261 (243-333)	212 (169-276)	335 (310-360)	255 (221-283)	251 (215-317)	>0.05
Serum creatinine (umol/l)		67.00 (62.6-73.8)		71.15 ± 18.2	68.62 ± 13.8	67.2 (61.9-72.9)	62.0 (60.6-63.3)	76.38 ± 25.0	68.3 (59.2-70.0)	>0.05
Total protein (g/l)		69.0 (67.0-70.7)		70.4 ± 5.1	70.0 ± 6.5	69.5 (67.5-70.1)	70.9 (68.8-73.0)	71.0 (67.4-74.0)	67.0 (65.0-72.6)	>0.05
Total bilirubin (umol/l)		11.5 (8.2-12.0)		13.2 ± 8.1	11.9 ± 6.0	10.8 (10.3-13.7)	11.5 (8.2-12.0)	16.6 ± 11.3	10.3 (8.5-11.8)	>0.05
ALP (U/l)		58.5 (52.5-70.7)		66.9 ± 20.5	70.1 ± 24.5	63.5 (39.6-69.6)	58.5 (55.0-75.0)	68.0 ± 20.1	62.1 (56.8-80.5)	>0.05
LDH (U/l)		171.0 (151.0-195.0)		188.5 (167.0-213.0)	191.4 ± 38.0	170.5 (146.0-233.0)	173.5 (159.0-188.0)	199.0 (181.0-221.0)	185.0 (176.0-201.0)	**<0.05**
IgG (g/l)		11.95 (10.9-13.1)		11.75 ± 3.2	12.5 ± 3.6	10.0 (8.3-11.5)	11.6 (9.0-14.2)	11.9 ± 3.0	9.8 (8.2-11.0)	>0.05
IgM (g/l)		1.5 (1.2-1.7)		1.63 ± 2.3	1.6 ± 2.2	0.69 ± 0.3	3.8 (1.1-6.6)	2.0 ± 2.8	0.9 (0.8-1.0)	**<0.05**
IgA (g/l)		1.8 (1.7-2.4)		2.32 ± 1.1	2.3 ± 1.0	2.0 ± 1.1	2.1 (1.6-2.6)	2.6 ± 1.3	2.1 (1.7-2.3)	>0.05
CRP (mg/l)		4.2 ± 5.2		4.0 ± 8.3	4.2 ± 11.7	1.5 ± 1.0	3.5 (2.9-4.1)	4.0 ± 5.5	6.4 ± 7.7	>0.05
Sodium (mmol/l)		140.0 (139.0-141.0)		140.0 ± 5.5	139.0 ± 8.8	141.0 (140.0-143.0)	139.5 (139.0-140.0)	140.0 (139.0-143.0)	140.0 (139.0-143.0)	>0.05
Potassium (mmol/l)		4.5 (4.4-4.7)		4.5 (4.2-4.7)	4.6 (4.4-4.9)	4.7 (4.5-4.9)	4.1 (4.0-4.1)	4.4 (4.1-4.6)	4.2 (4.0-4.5)	>0.05
Chlorides (mmol/l)		105.0 (104.0-106.0)		104.7 ± 2.3	104.5 (103.0-106.0)	106.0 (103.0-106.0)	104.0 (104.0-106.0)	104.5 (103.0-106.0)	105.0 (103.0-106.0)	>0.05
Vitamin D (ng/ml)		34.0 (23.6-40.9)		31.7 ± 15.3	28.9 (21.1-44.8)	35.7 (24.6-46.6)	18.9 (7.9-29.9)	25.9 (16.7-35.6)	32.4 (18.6-44.1)	>0.05
ASPAT (U/l)		20.7 ± 6.9		21.5 ± 6.2	22.6 ± 7.7	20.0 (18.0-22.0)	17.0 (16.0-18.0)	22.0 (17.0-26.0)	20.0 (18.0-21.0)	>0.05
ALAT (U/l)		22.3 ± 15.5		22.4 ± 11.9	21.7 ± 9.0	19.0 (17.0-23.0)	16.5 (13.0-20.0)	20.0 ± 16.9	17.0 (15.0-22.0)	>0.05
Albumin (g/l)		9.2 ± 13.0		10.1 ± 12.8	14.0 ± 16.2	8.6 ± 11.6	19.9 (4.4-35.4)	5.9 ± 16.2	8.8 ± 11.8	>0.05
CA15-3		15.9 (11.9-17.4)		63.95 ± 310.0	142.5 ± 509.6	23.6 (6.8-26.0)	15.6 (13.5-17.7)	18.2 ± 10.17	20.2 (15.3-30.0)	>0.05

Data are presented as mean ± standard deviation in case of normally distributed data and median and interquartile range in non-normally distributed data. The asterisk symbol (*) means reference group vs. study group. Statistically significant values are shown in bold.

### 2.2 Serum preparation

Blood was collected from patients in the morning from the cubital vein into a tube containing a clotting activator to obtain serum. Thirty minutes after collection, the tubes were centrifuged at 1500 x g for 15 minutes at room temperature. The serum obtained was separated from the clot, frozen at -80 °C, and stored in this manner until analysis.

### 2.3 ELISA tests

Serum adipokines concentrations were determined by enzyme-linked immunosorbent assay (ELISA) using the kits presented in **Table 3**.

**Table 3:** Tests performed and their specifications.

Parameter	Test name	Producer	Test method	Detection range	Sensitivity	Precision	
						Intra-Assay	Inter-Assay
Progranulin	Progranulin Human ELISA (RMEE103)	BioVendor	Sandwich ELISA	75–2500 pg/mL*	0.018 ng/ml	CV>8,0%	CV>4,4%
FSTL1	ELISA Kit for Follistatin Like Protein 1 (FSTL1) (SEJ085Hu)	Cloud-Clone Corp.	Double-antibody Sandwich	1.56–100 ng/mL	<0.59 ng/ml	CV<10%	CV<12%
Asprosin	ELISA Kit for Asprosin (Asp) (SEA332Hu)	Cloud-Clone Corp.	Double-antibody Sandwich	0.156–10 ng/mL	<0.064 ng/ml	CV<10%	CV<12%
Meteorin	ELISA Kit for Meteorin (METRN) (SEH662Hu)	Cloud-Clone Corp.	Double-antibody Sandwich	0.156–10 ng/mL	<0.057 ng/ml	CV<10%	CV<12%
Adipsin	ELISA Kit for Complement Factor D (CFD) (SEB833Hu)	Cloud-Clone Corp.	Double-antibody Sandwich	0.625–40 ng/mL	<0.238 ng/ml	CV<10%	CV<12%
Nesfatin	ELISA Kit for Nesfatin 1 (NES1) (CEA242Hu)	Cloud-Clone Corp.	Competitive Inhibition	617.3–50000 pg/mL	<234.2 pg/ml	CV<10%	CV<12%
Neuregulin 4	ELISA Kit for Neuregulin 4 (NRG4) (SEC174Hu)	Cloud-Clone Corp.	Double-antibody Sandwich	0.156–10 ng/mL	<0.056 ng/ml	CV<10%	CV<12%
Isthmin 1	ELISA Kit for Isthmin 1 (ISM1) (SEQ515Hu)	Cloud-Clone Corp.	Double-antibody Sandwich	0.156–10 ng/mL	<0.064 ng/ml	CV<10%	CV<12%

* Calibration range.

### 2.4 Statistical analysis

The obtained results were statistically analyzed using Statistica 13.3 (StatSoft Polska Sp. z o.o.). The Shapiro-Wilk test was used to verify normality of distribution. In cases where the distribution of the analyzed parameters was non-normal, a non-parametric Kruskal-Wallis ANOVA and Mann-Whitney U test was performed. When p<0.05, a *post-hoc* test was performed – Tukey’s test for unequal sample sizes. The results obtained were presented using a box plot. However, when the distribution of the analyzed parameters was normal, a parametric ANOVA test was performed. When p<0.05, a *post-hoc* test was performed. The results obtained were presented using a box plot. The association between parameters was investigated using Spearman’s correlation analysis.

## 3 Results

The results of adipokine concentrations in patients from the control group and patients from the study group are presented in **Table 4**.

**Table 4:** Basic descriptive statistics of the analyzed parameters.

Parameters	Group	Molecular breast cancer subtype	Statistical parameters							
			N	m	s	Me	Q_1_	Q_3_	x_min_	x_max_
PGRN (pg/ml)	Study group	Total	59	1693.302	355.606	1652.190	1416.757	1936.672	889.933	2459.489
		Luminal A	21	1622.971	407.193	1616.267	1395.617	1865.103	889.933	2426.053
		Luminal B HER2+	8	1736.626	370.077	1677.820	1385.117	2095.286	1342.700	2233.865
		Non-luminal B HER2+	2	1713.881	567.329	1713.881	1312.718	2115.043	1312.718	2115.043
		Luminal B HER2-	23	1719.507	325.298	1662.276	1506.150	1892.874	1212.963	2459.489
		TNBC	5	1790.604	238.137	1796.849	1700.410	1914.566	1452.403	2088.792
	Reference group	–	13	1906.733	323.511	1863.997	1771.288	2133.143	1336.206	2458.660
FSTL1 (ng/ml)	Study group	Total	64	<LLOQ	–	–	–	–	–	–
		Luminal A	23	<LLOQ	–	–	–	–	–	–
		Luminal B HER2+	9	<LLOQ	–	–	–	–	–	–
		Non-luminal B HER2+	2	<LLOQ	–	–	–	–	–	–
		Luminal B HER2-	23	<LLOQ	–	–	–	–	–	–
		TNBC	7	<LLOQ	–	–	–	–	–	–
	Reference group	–	16	<LLOQ	–	–	–	–	–	–
Asprosin (ng/ml)	Study group	Total	64	1.001	0.289	0.961	0.798	1.173	0.455	1.607
		Luminal A	23	0.956	0.297	0.895	0.803	1.171	0.455	1.588
		Luminal B HER2+	9	0.947	0.255	0.932	0.770	1.087	0.625	1.430
		Non-luminal B HER2+	2	1.004	0.094	1.004	0.937	1.070	0.937	1.070
		Luminal B HER2-	23	1.070	0.314	1.001	0.793	1.378	0.596	1.607
		TNBC	7	0.992	0.276	0.947	0.740	1.174	0.712	1.496
	Reference group	–	16	0.890	0.271	0.856	0.726	1.146	0.336	1.410
METRN (ng/ml)	Study group	Total	64	>ULOQ	–	–	–	–	–	–
		Luminal A	23	>ULOQ	–	–	–	–	–	–
		Luminal B HER2+	9	>ULOQ	–	–	–	–	–	–
		Non-luminal B HER2+	2	>ULOQ	–	–	–	–	–	–
		Luminal B HER2-	23	8.852	–	–	–	–	–	–
		TNBC	7	8.148	–	–	–	–	–	–
	Reference group	–	16	>ULOQ	–	–	–	–	–	–
CFD (ng/ml)	Study group	Total	62	3.134	1.479	2.831	2.147	4.042	0.692	7.265
		Luminal A	22	3.128	1.742	2.443	2.005	4.635	0.860	6.465
		Luminal B HER2+	9	2.864	1.402	2.861	2.157	3.525	0.692	5.684
		Non-luminal B HER2+	2	5.107	3.053	5.107	2.948	7.265	2.948	7.265
		Luminal B HER2-	22	3.101	1.079	2.937	2.208	4.042	1.209	4.848
		TNBC	7	3.038	1.400	2.801	1.843	4.169	1.072	5.106
	Reference group	–	15	2.282	1.199	2.152	1.209	3.236	0.743	4.564
Nesfatin (pg/ml)	Study group	All	62	13457.05	6331.182	14537.98	7546.995	17610.93	665.416	25590.39
		Luminal A	22	13427.24	6043.474	13574.98	7546.995	17561.88	3462.944	25590.39
		Luminal B HER2+	9	12143.94	7498.952	9659.760	8307.213	18818.22	1616.161	23670.04
		Non-luminal B HER2+	2	10309.62	4296.442	10309.62	7271.581	13347.67	7271.581	13347.67
		Luminal B HER2-	22	12775.75	6154.070	14432.35	7331.946	16556.43	665.416	23737.95
		TNBC	7	18279.52	5860.983	19491.67	14634.19	23824.72	7371.560	24484.96
	Reference group	–	16	13744.52	4609.918	12932.66	11161.33	15606.63	6126.537	22815.50
NRG4 (ng/ml)	Study group	All	64	<LLOQ	–	–	–	–	–	–
		Luminal A	23	<LLOQ	–	–	–	–	–	–
		Luminal B HER2+	9	<LLOQ	–	–	–	–	–	–
		Non-luminal B HER2+	2	<LLOQ	–	–	–	–	–	–
		Luminal B HER2-	23	<LLOQ	–	–	–	–	–	–
		TNBC	7	<LLOQ	–	–	–	–	–	–
	Reference group	–	16	<LLOQ	–	–	–	–	–	–
ISM1 (ng/ml)	Study group	All	64	2.807	1.245	2.674	2.330	2.963	1.617	11.921
		Luminal A	23	2.569	0.505	2.544	2.156	2.801	1.617	3.959
		Luminal B HER2+	9	2.876	0.316	2.899	2.623	3.012	2.468	3.416
		Non-luminal B HER2+	2	2.695	0.293	2.695	2.488	2.902	2.488	2.902
		Luminal B HER2-	23	2.963	2.004	2.547	2.135	2.924	1.966	11.921
		TNBC	7	3.017	0.322	3.103	2.742	3.239	2.484	3.457
	Reference group	–	16	2.974	0.432	3.063	2.858	3.252	1.904	3.495

N, abundance; m, mean; s, standard deviation; Me, median; Q_1_, lower quartile; Q_3_, upper quartile; x_min_, minimum value; x_max_, maximum value; BLOQ, Below the Limit of Quantification; LLOQ, Lower Limit of Quantification; ULOQ, Upper Limit of Quantification.

Due to the non-obtainment of results within the range of detectability for the FSTL1, METRN, and NRG adipokine tests, they were excluded from further statistical analysis.

### 3.1 Adipokines concentrations in the serum of women with benign breast lesions and in the serum of women with breast cancer

**Figure 1:**
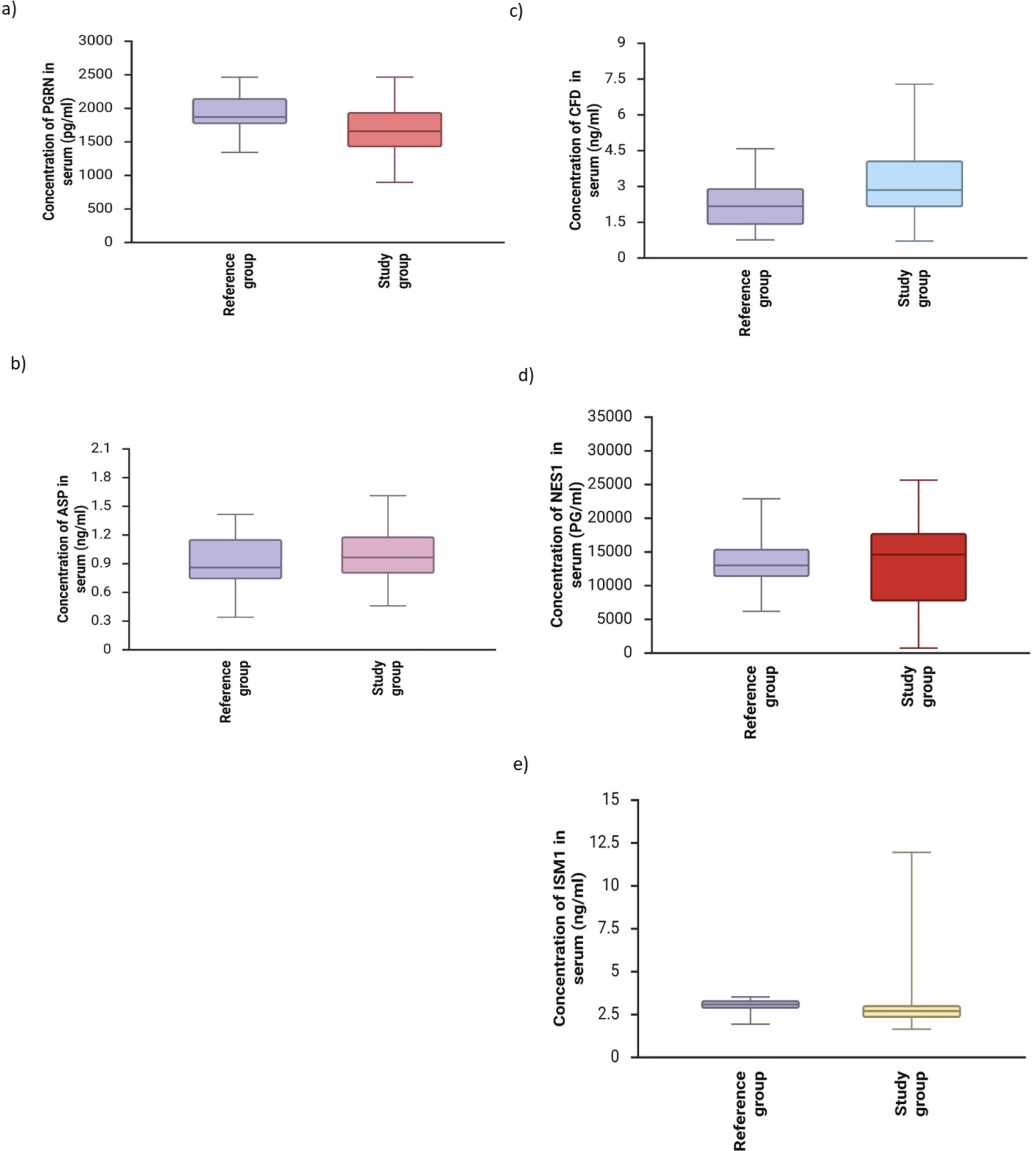
Concentrations of adipokines [**(a)**-PGRN, **(b)**-Asp, **(c)**-CFD, **(d)**-NES1, **(e)**-ISM1] in the serum of women with benign breast lesions and in the serum of women with breast cancer (p<0.05).

**Figure 1** presents adipokine concentrations in the serum of women with benign breast lesions and in the serum of women with breast cancer.

A statistically significant difference was found between the concentrations of CFD and ISM1 in the serum of breast cancer patients compared to the serum concentrations of patients in the control group (p<0.05). No statistically significant differences were found between the serum concentrations of PGRN, Asp, NES1, and NRG4 in breast cancer patients compared to the serum concentrations in the reference group. Considering the division of breast cancer into luminal and non-luminal breast cancers, a statistically significant difference was found between the serum concentrations ISM1 in luminal breast cancer patients compared to serum concentrations in patients from the reference group (p<0.05).

### 3.2 Adipokines concentrations in the serum of women with breast cancer with different degrees of malignancy

**Figure 2:**
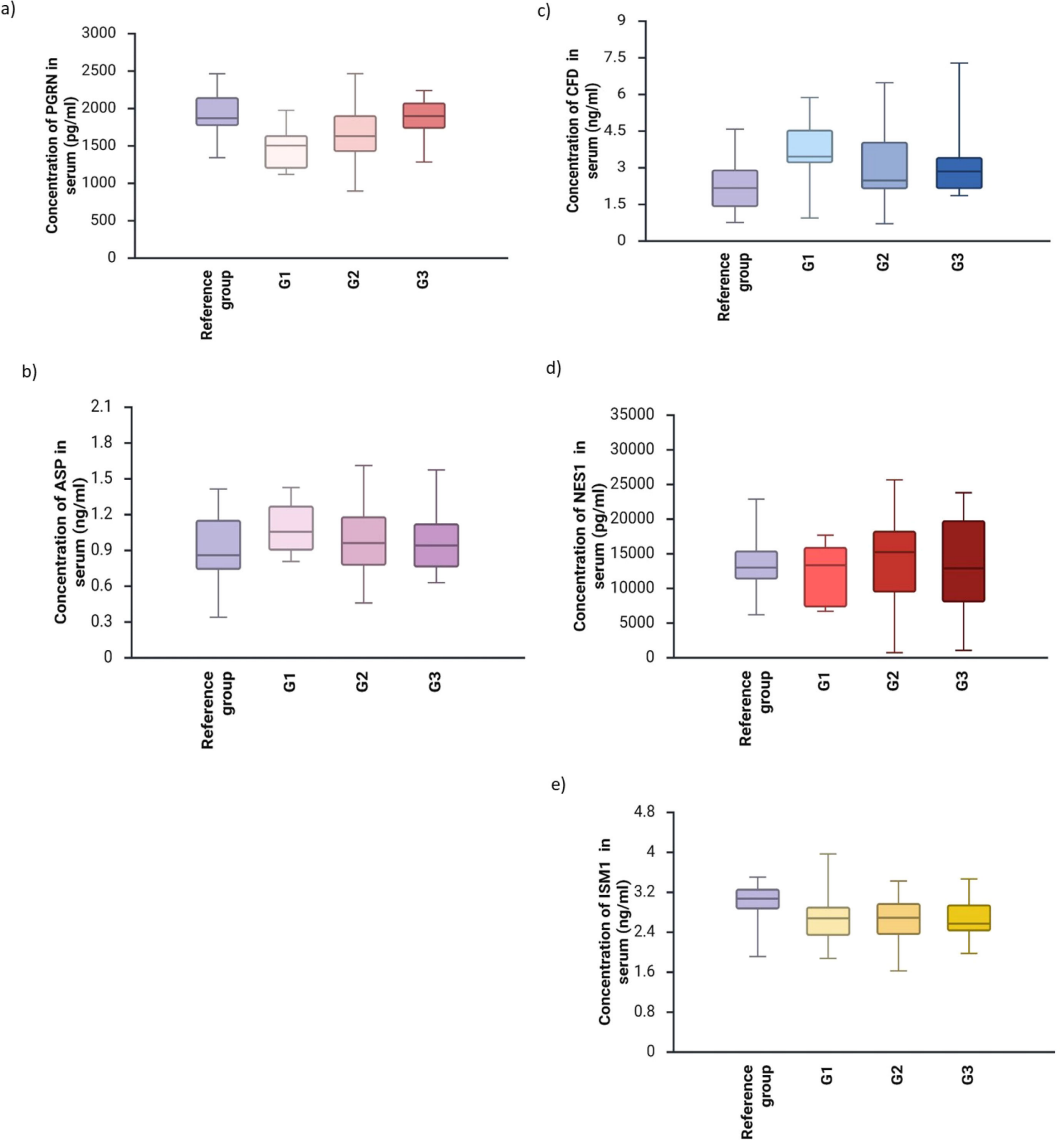
Concentrations of adipokines [**(a)**-PGRN, **(b)**-Asp, **(c)**-CFD, **(d)**-NES1, **(e)**-ISM1] in the serum of women with breast cancer with different degrees of malignancy (p<0.05).

**Figure 2** presents the concentrations of adipokines in the serum of women with breast cancer with different degrees of malignancy.

No statistically significant differences were found between serum concentrations of PGRN, Asp, CFD, NES1, and ISM1 in patients with different degrees of breast cancer malignancy.

### 3.3 Adipokines concentrations in the serum of women with breast cancer with different molecular tumor subtypes

**Figure 3:**
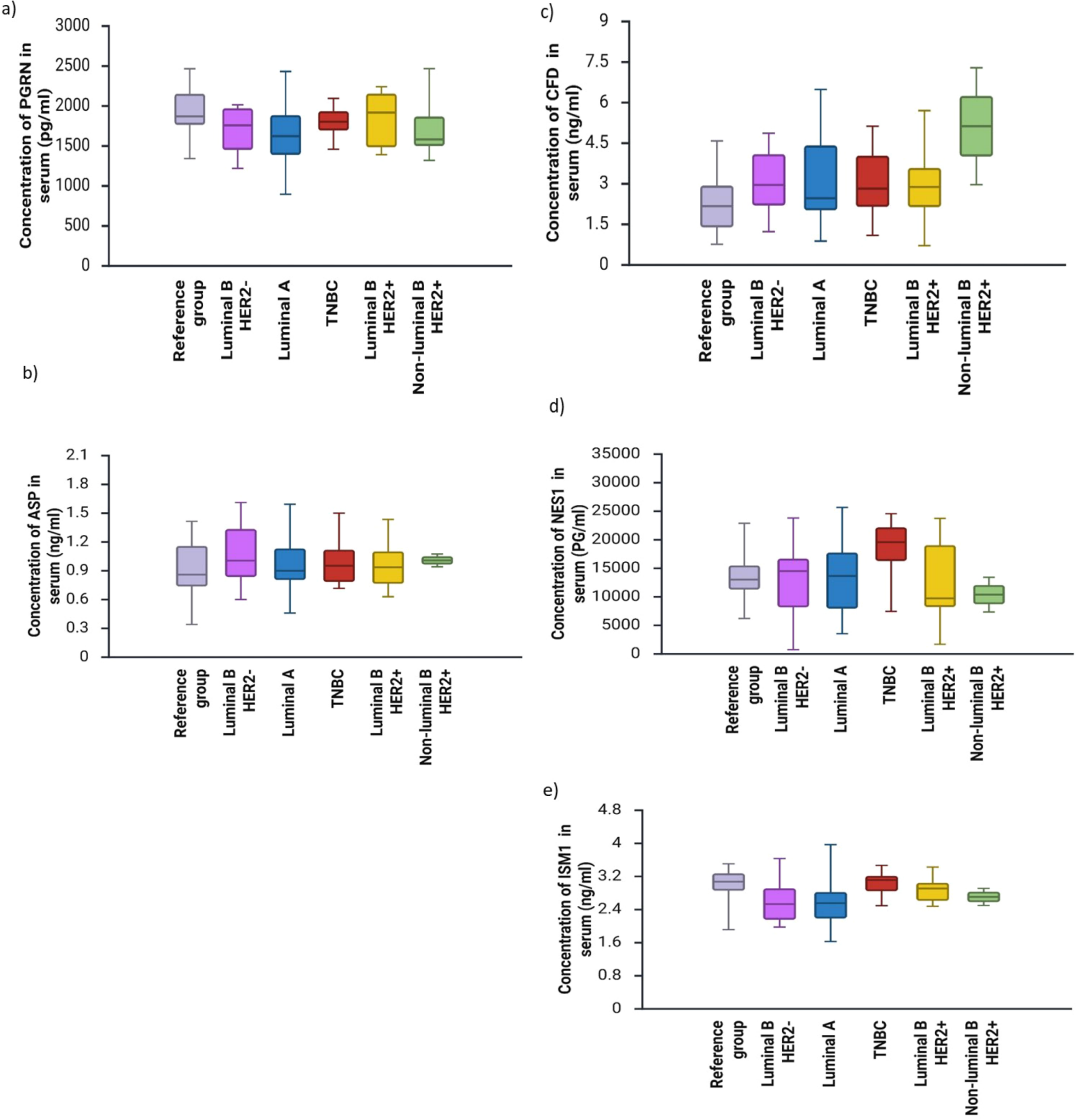
Concentrations of adipokines [**(a)**- PGRN, **(b)**-Asp, **(c)**-CFD, **(d)**-NES1, **(e)**-ISM1] in the serum of women with breast cancer with different molecular tumor subtypes (p<0.05).

**Figure 3** presents the concentrations of adipokines in the serum of women with breast cancer with different molecular tumor subtypes.

No statistically significant differences were found between the concentrations of PGRN, Asp, CFD, NES1, and ISM1 in the serum of breast cancer patients compared to the concentrations in the reference group.

### 3.4 Adipokines concentrations in the serum of women from the reference group and women from the study group depending on BMI

**Table 5** presents the concentrations of the studied adipokines depending on BMI.

**Table 5:** Concentrations of the adipokines studied in the reference and study group depending on the BMI.

Parameters	Reference group		*p* value *	Study group		*p* value *
	BMI ≤ 24,99	BMI>24,99		BMI ≤ 24,99	BMI>24,99	
PGRN (pg/ml)	1904.01 ± 353.563	1834.29 (1748.08 - 1854.0)	p>0,05	1617.47 ± 280.817	1648.15 ± 381.786	p>0,05
Asp (ng/ml)	0.96 ± 0.310	0.85 ± 0.184	p>0,05	0.99 ± 0.265	1.02 ± 0.292	p>0,05
CFD (ng/ml)	1.65 ± 0.666	2.29 ± 0.962	p>0,05	3.29 ± 1.391	2.74 ± 1.136	p>0,05
NES1 (pg/ml)	14746.62 ± 5836.610	12435.12 ± 3262.635	p>0,05	12966.77 ± 7662.861	12086.52 ± 5465.775	p>0,05
ISM1 (ng/ml)	2.85 ± 0.628	3.02 ± 0.378	p>0,05	2.61 ± 0.522	2.64 ± 0.445	p>0,05

Data are presented as mean ± standard deviation in case of normally distributed data and median and interquartile range in non-normally distributed data. The asterisk symbol (*) means BMI ≤ 24,99 vs. BMI>24,99.

### 3.5 Correlation of other parameters with adipokines

The association between adipokines and other parameters: BMI, T (tumor), N (lymph node), M (metastasis), WBC (White Blood Cells), HGB (Hemoglobin), PLT (Platelets), creatinine, total protein, total bilirubin, ALP (Alkaline Phosphatase), LDH (Lactate Dehydrogenase), IgG (Immunoglobulin G), IgM (Immunoglobulin M), IgA (Immunoglobulin A), CRP (C Reactive Protein), sodium, potassium, chloride, vitamin D, ASPAT (Aspartate Aminotransferase), ALAT (Alanine aminotransferase) and albumin was investigated using Spearman’s correlation analysis. The correlations are presented in **Table 6**.

**Table 6:** Positive and negative correlations of other parameters with adipokines.

Adipokin	Positive correlation	Negative correlation
Reference group & Study group		
PGRN	sodium*, CA15-3*	chlorides***
Asp	BMI*, M**, HGB**, total protein**, sodium**, potassium**, ALAT**	WBC*, chlorides*, albumin*, CA15-3*
CFD	NES1*, M*, PLT***, ALP*, IgM*	sodium*, vitamin D**, ASPAT*, ALAT*
NES1	ISM1**, WBC**, serum creatinine*, CRP*	potassium*
ISM1	NES1**, BMI*, PLT*, sodium**, potassium*	IgG*
Study group		
PGRN	PLT*, ALAT*	sodium***, chlorides***
Asp	M**, HGB*, total bilirubin*, potassium*, CA15-3**	NES1*, WBC*
CFD	M*, PLT*, vitamin D*,	HGB*, ALP*, ASPAT*
NES1	HGB*, serum creatinine*, IgA**, CRP**, potassium*	Asp*
ISM1	PLT*	LDH*, sodium*, potassium*
Reference group		
Asp	sodium*,	ISM1*, PLT*, IgG**
CFD	PLT*, ALP*, sodium*, chlorides*	IgA*, vitamin D*
NES1	CA15-3*	serum creatinine*, LDH**
ISM1	CRP*, albumin*	Asp*, total bilirubin*
Group of patients with G1 malignancy		
PGRN	IgG*	serum creatinine*
Asp	PLT*	IgM*
CFD	HGB*	
NES1	ASPAT*, ALAT*	PLT*, serum creatinine*
Group of patients with G2 malignancy		
PGRN	N*, IgA**, ALAT*, CA15-3*	T**, M*
Asp	N**, chlorides**, vitamin D*	NES1*, M*, sodium*
CFD	CA15-3*	T*, HGB*, CRP*, ASPAT*
NES1	N*	Asp*, WBC*, ALP*, IgG**, IgM*, CRP*, CA15-3*
ISM1	WBC*, IgM*, chlorides**, ASPAT*	ALAT**
Group of patients with G3 malignancy		
PRGN	serum creatinine*	LDH*
Asp	IgG*	sodium*
CFD		BMI*, ALAT*
NES1	sodium*, vitamin D*	
ISM1		N*
Group of patients with luminal A cancer		
PGRN	T*, CRP*, potassium*	ALP*
Asp	serum creatinine*, total bilirubin*, IgA*	CFD*, total protein*, chlorides*, vitamin D*
CFD	vitamin D**	Asp*, ALAT*
NES1	total protein*, IgM*, CRP*	serum creatinine**
ISM1	WBC*	HGB*, LDH*
Group of patients with luminal B HER+ cancer		
PGRN	CA15-3*	
Asp	PLT*, ALP*	vitamin D*
NES1	total protein*	ISM1*
ISM1	CA15-3*	NES1*
Group of patients with luminal B HER- cancer		
PRGN	total bilirubin*, CA15-3*	ISM1**, sodium*, chlorides*
Asp	LDH**, IgG*, ALAT*	total bilirubin*
CFD	IgG*	NES1*, T*, sodium*, vitamin D*, ALAT*
NES1	total protein**, ALP*	CFD*, IgG*, vitamin D*
ISM1	LDH**	PGRN**, IgA*
Group of patients with TNBC		
Asp	CFD*	LDH*
CFD	Asp*	
ISM1		IgG*

*p<0.05, **p<0.01, ***p<0.001.

**Figure 4:**
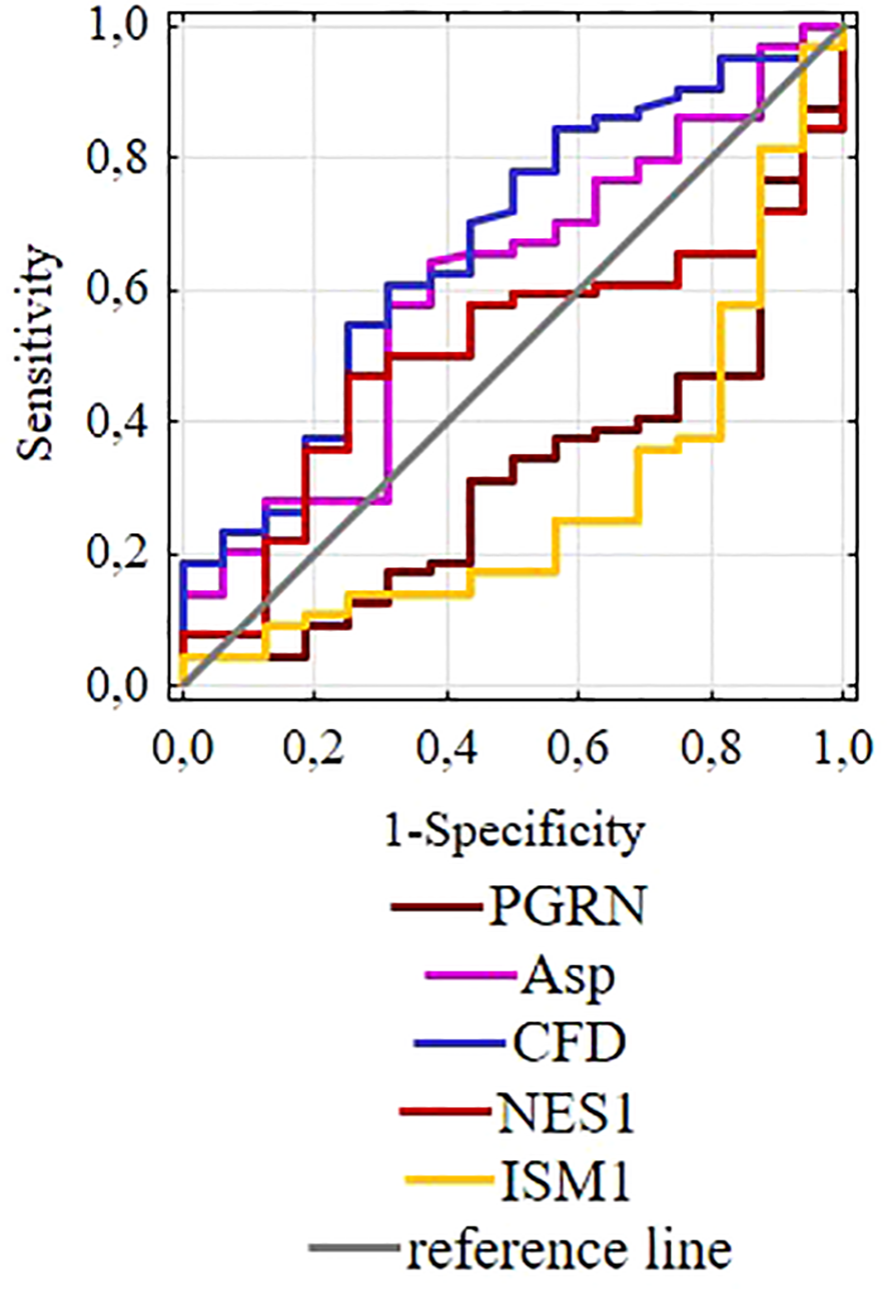
ROC curves for selected adipokines.

Along with the obtained ROC curves, presented in **Figure 4**, AUC values were obtained, which do not indicate the potential diagnostic usefulness of the tested parameters, at least when used individually.

**Figure 5:**
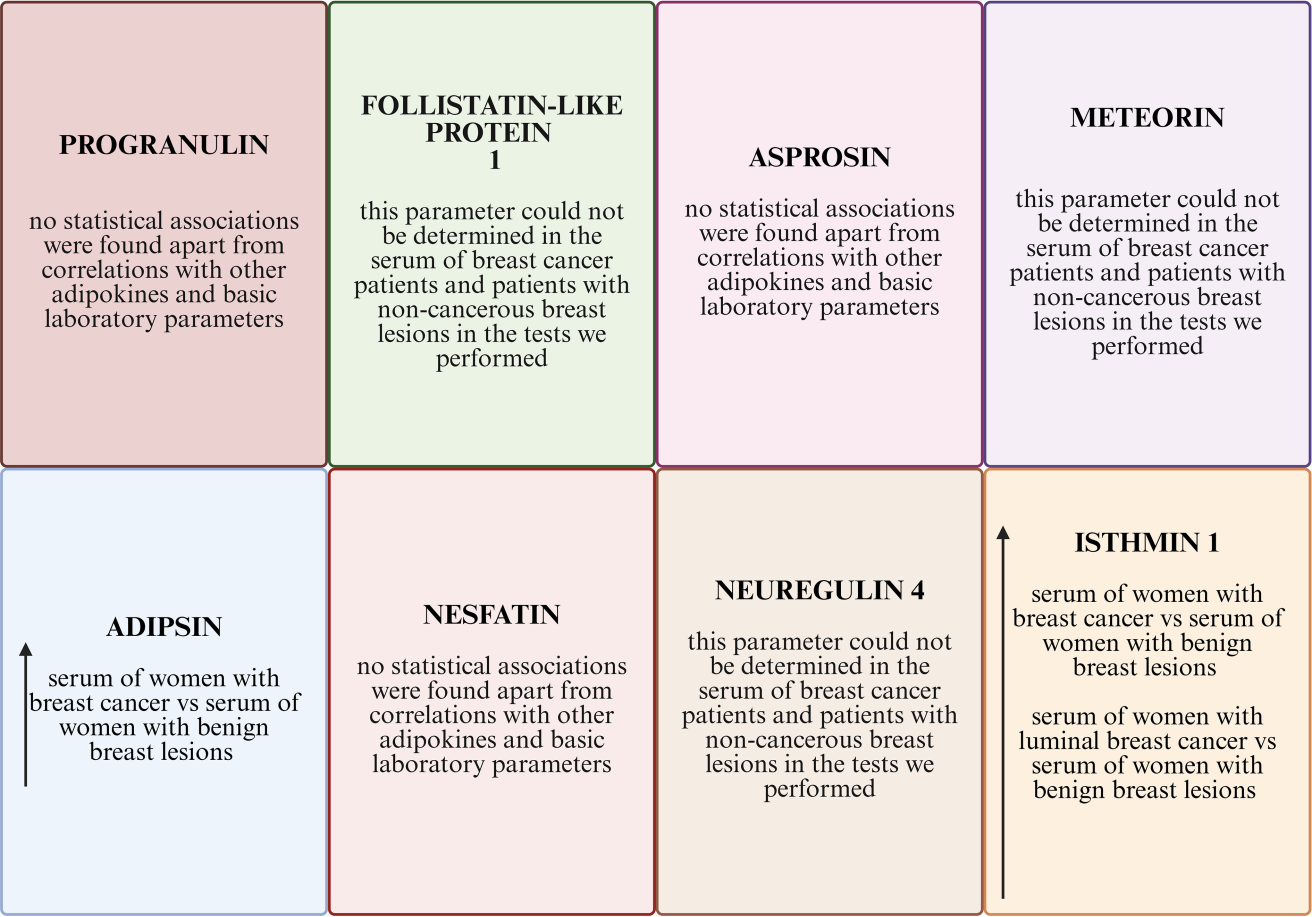
Summary of results.

**Figure 5** presents a summary of the results obtained.

## 4 Discussion

Adipokines play diverse roles in the initiation, progression, metastasis, and treatment response of breast cancer (13). Both the concentration and expression of adipokines and their receptors are dysregulated in breast cancer (13). Numerous studies on **progranulin** in breast cancer have reported its role in various pathways of activity related to carcinogenesis and its correlation with unfavorable breast cancer diagnosis. Li et al. (55) demonstrated overexpression of progranulin in breast cancer, which correlated with tumor size, lymph node metastasis, and TNM stage. High progranulin expression was also associated with higher tumor angiogenesis, as reflected by increased VEGF expression and higher microvessel density (55). In IDC, higher progranulin expression was found than in DCIS (55). A study by Li et al. (30) did not show differences in progranulin expression in individual breast cancer subtypes, but the researchers observed an upward trend in the TNBC group. Berger et al. (56) reported that inhibition of PGRN binding to its main receptor, sortilin, can block PGRN-induced TNBC metastasis. Co-expression of PGRN and sortilin is a prognostic biomarker identifying more aggressive breast cancer (32). A study by Serrero & Ioffe (57) showed that progranulin is almost never expressed in benign breast epithelium. In a study by Zhou et al. (36), PGRN was overexpressed in breast cancer tissues compared to paraneoplastic tissues. PGRN expression, which is elevated in 30% of TNBC cases, is an independent prognostic factor for recurrence in tissue, while elevated serum PGRN levels are associated with poor prognosis (58). Inhibition of PGRN expression by silencing RNA (siRNA) and treatment with anti-progranulin antibody (AG01) reduced proliferation and migration in a dose-dependent manner in breast cancer cells (58). Koo et al. (43, 59) reported that serum PGRN concentration correlates with recurrence in patients with HR-positive breast cancer during adjuvant tamoxifen therapy and that preoperative serum PGRN concentration was clinically significant in predicting cancer mortality in breast cancer patients, regardless of disease stage and metabolic parameters, especially in HR-positive tumors. In these studies, the mean PGRN concentration in HR-positive patients was 122 (98–105) ng/ml, whereas in HR-negative patients it was 127 (105–153) ng/ml (59). Higher PGRN concentrations were associated with an increased risk of death from breast cancer (59). In a study by Tkaczuk et al. (60), serum PGRN concentrations were higher in breast cancer patients compared to the serum of healthy women. The researchers also observed a difference between disease stages, with serum PGRN concentrations higher in stage 4 patients compared to stage 1–3 patients (60). However, no differences were observed between PGRN concentration and age, race, tumor stage, estrogen receptor (ER) expression, PR receptor expression, or HER-2 receptor expression (60). Another study by the same group of researchers on patients with metastatic breast cancer showed that serum PGRN concentrations in relation to disease status have a statistically significant association with disease progression or response to treatment (61). Patients with serum PGRN concentrations <55 ng/mL had a four-fold longer survival compared to patients with PGRN concentrations above this range (61). Contrary to the findings of the majority of studies referenced, no differences in serum progranulin concentrations were observed between women diagnosed with breast cancer and those with non-cancerous breast lesions. It is possible that a difference may be observed if the control group consists of healthy women.

Regarding **follistatin-like protein 1 (FSTL1)**, most studies conducted to date have focused on evaluating the expression of this adipokine. Jin et al. (62) found that miR-524-5p targeting FSTL1 adversely affects the progression of migration, invasion, and EMT of breast cancer cells. Yang et al. (44) found that FSTL1 expression was significantly low in breast cancer tissues compared to normal breast tissues and that high FSTL1 expression in patients indicated prolonged survival. In all stages of invasive breast cancer, there was no FSTL1 expression compared to normal breast tissue (44). The same result was observed for various stages of nodal metastasis (44). In addition, invasive breast cancer tissues, segregated by luminal tissue, HER2, and TNBC, showed lower FSTL1 expression than normal tissues (44). The number of ALCAM+ cells, i.e. bone marrow-derived pluripotent mesenchymal stem cells that induce bone metastases, increased with a positive result for FSTL1 in advanced breast cancer tissues, but not in adjacent healthy tissues (45). In a study by An et al. (48), increased FSTL1 expression was associated with inhibited cell proliferation in the MDA-MB-231-BR metastatic cell line compared to the MDA-MB-231 parental line. Interestingly, this protein was almost undetectable in the other breast cancer cell lines tested (48). Research by Cheng et al. (47) demonstrated significantly higher levels of FSTL1 mRNA and protein in TNBC cells than in non-TNBC samples and breast cancer cell lines. Higher levels of FSTL1 were also found in chemo-resistant cells (47). Overexpression of FSTL1 resulted in a significant increase in breast cancer cell proliferation (47). In a study by Zhang et al. (63), FSTL1 expression was reduced in both breast cancer tissue and serum of breast cancer patients. Decreased FSTL1 expression was associated with reduced survival in breast cancer patients, and studies in mice showed that FSTL1 deficiency did not affect primary tumor growth but increased breast cancer cell metastasis to the lungs, resulting in reduced survival of tumor-bearing mice (63). The researchers also found no correlation between FSTL1 and ER, PR, HER2, grade 1–2 tumors, or T status (63). The results obtained by other researchers indicating that FSTL1 expression in breast cancer is low or virtually absent correspond to our results, i.e., FSTL1 concentration was undetectable in the serum of breast cancer patients and the reference group.

**Asprosin** immunoreactivity in IDC tissues was higher compared to healthy breast tissues (64). No difference in asprosin immunoreactivity was detected between the three degrees of tumor malignancy (64). Kocaman et al. (65) also observed higher asprosin immunoreactivity in IDC tissues compared to healthy breast tissues. Yur et al. (66) observed higher serum asprosin concentrations in women with breast cancer compared to serum concentrations in healthy individuals (66). This parameter showed a sensitivity of 0.825 and a specificity of 0.750 (66). The researchers found no difference between breast cancer stages and asprosin concentration (66). Regarding asprosin, we did not observe any statistically significant differences between the study group and the reference group, or between individual types of malignancy and molecular subtypes, which is partly consistent with the findings of other researchers.

The immunoreactivity of meteorin-like protein (METRNL), a protein with 46% amino acid sequence homology to **meteorin**, was higher in IDC tissues compared to normal breast tissues (65, 67). To our knowledge, we are currently the only group of researchers to have performed the measurement of meteorin level in the serum of breast cancer patients and women with benign breast lesions.

The findings of Rajkumar et al. (68) indicate reduced expression of **adipsin** in breast cancer tissues and reduced plasma concentrations of this adipokine compared to the control group. The combination of adipsin concentration with other protein markers: leptin, syndecan-1, basic fibroblast growth factor, interleukin 17B, and Dickopff-3 in the detection of breast cancer was characterized by a sensitivity of 65% and a specificity of 80% (68). Goto et al. (51) detected adipsin expression both in adipose tissue from surgical specimens of breast cancer patients and in ADSCs isolated from them, and its expression level was significantly higher in obese patients. In a study conducted by Sat-Muñoz et al. (11), in women with a BMI below 25 with benign breast lesions, the median and range between quartiles 1 and 3 was 0.24 μg/ml (0.02–1.3), in women with breast cancer 0.9 μg/ml (0.00–1.3), and in healthy women 0.35 μg/ml (0.25–0.48). For women with a BMI above 25, the results were as follows: in women with benign breast changes, 1.04 μg/ml (0.09–1.5); in women with breast cancer, 0.9 μg/ml (0.06–1.3), and in healthy women 0.47 μg/ml (0.4–0.56). Our study showed a statistically significant difference between serum adipsin concentrations in breast cancer patients compared to the reference group, which may indicate systemic immune and inflammatory response disorders involving the adipokine under study.

**Nesfatin** is produced by the cleavage of nucleobindin-2 (NUCB2), and since they are located in the same place, these names are used interchangeably (52, 69). In a study by Suzuki et al. (53), NUCB2 expression was increased by estradiol in estrogen receptor-positive breast cancer cells. NUCB2 immunoreactivity was detected in approximately half of the breast cancer tissues examined and was positively associated with lymph node metastasis and ER status in patients (53). NUCB2 status was also significantly associated with an increased risk of recurrence and poor clinical outcomes in patients (53). Kmiecik et al. (69) observed a statistically significantly higher level of NUCB2/NESF-1 in IDC cells compared to mastopathy samples. Analysis of the five-year survival rate showed that positive expression of NUCB2/NESF-1 in cancer cells was also associated with longer patient survival (69). NUCB2 expression in breast tumors was weakly positively correlated with patient age, with higher protein expression in elderly patients (69). No correlation was found between positive NUCB2 expression and tumor size, stage, HER2 protein, or lymph node metastasis, whereas in our study, serum NES1 concentration in patients with luminal A cancer was positively correlated with T, i.e., tumor size (69). The researchers found an inverse relationship between NUCB2 and increasing malignancy of breast cancer cells, with the lowest expression levels found in poorly differentiated breast cancer cells (G3) (69). NUCB2 expression was significantly lower in TNBC compared to other breast cancer samples (69). In a study by Zeng et al. (70), NUCB2 protein concentration decreased in paired metastatic lymph nodes, with a positive expression rate of 82% in primary breast cancer tissues and 47% in paired metastatic lymph nodes, respectively. In a study by Ning et al. (52), the mean serum nesfatin-1 concentration in breast cancer patients was significantly higher than in healthy individuals, while in our study, the serum concentration in breast cancer patients and patients with non-cancerous breast lesions was comparable.

Dunn et al. (71) detected **neuregulin 4** mRNA in breast cancer cell lines. NRG4 mRNA expression was demonstrated by *in situ* hybridization in sections of primary breast tumors (71). Marshall et al. (72) used immunohistochemical staining to demonstrate high levels of NRG4 expression in highly malignant pre-invasive ductal carcinoma *in situ* of the breast. Each of the neuregulins examined by the researchers was highly expressed in a significant proportion of tumors, showing a predominantly homogeneous cytoplasmic staining pattern (72). To our knowledge, we are currently the only group of researchers to have performed NRG4 testing in the serum of breast cancer patients and women with benign breast lesions.

In ductal breast cancer tissues, Turk et al. (25) observed decreased expression of isthmin. Suman et al. (73) examined the variability of DNA methylation levels across the genome in invasive lobular breast cancer (ILBC) tumors and assessed the relationship between methylation levels in regions with different methylation patterns and overall survival in women with ILBC. One of the regions with the highest methylation variability was located in the promoter region of the gene encoding **isthmin**, ISM1 (73). A weak association with shorter overall survival was observed for ISM1 (73). We were the first to measure ISM1 concentrations in the serum of breast cancer patients and women with benign breast lesions. Our studies showed a statistically significant difference between serum **isthmin** concentrations in breast cancer patients compared to the reference group, which may indicate systemic immune and inflammatory response disorders involving the studied adipokine.

In this study, for the first time, the serum concentrations of eight selected new adipokines were simultaneously measured: progranulin, follistatin-like protein 1, asprosin, meteorin, adipsin, nesfatin, neuregulin 4, and isthmin in breast cancer patients, but further studies on a larger research group seem necessary to better understand the role of these adipokines in the development of breast cancer and to determine their potential significance in diagnosis, prognosis, and treatment.

Interestingly, we demonstrated that the highest correlations occurred between the studied adipokines. PGRN in the study group and reference group correlated positively with CA15-3 (p<0.05) and negatively with chlorides (p<0.001). In the study group PGRN correlated positively with PLT and ALAT (p<0.05) and negatively with sodium and chlorides (p<0.001). In the group of patients with G1 malignancy, PGRN was positively correlated with IgG (p<0.05) and negatively correlated with serum creatinine (p<0.05), oppositely, in patients with G3 malignancy, a positive correlation with serum creatinine (p<0.05) and a negative correlation with LDH (p<0.05) were observed. In turn, in the group of patients with G2 malignancy, PGRN was positively correlated with N, ALAT, CA15-3 (p<0.05), IgA (p<0.01) and negatively correlated with M (p<0.05) and T (p<0.01). In patients with luminal A cancer, PGRN correlated with T, potassium and CRP (p<0.05), and that may be related to the fact that both molecules are associated with inflammation, which is associated with the cancer. In patients with HER+ luminal B tumors, PGRN correlated with CA15-3, a breast cancer marker (p<0.05).In patients with luminal B HER- cancer, PGRN is positively correlated with total bilirubin and CA15-3 (p<0.05), which is particularly interesting considering that bilirubin is an endogenous antioxidant with anti-inflammatory properties, and negatively correlated with ISM1 (p<0.01), sodium, and chlorides (p<0.05) (74).

In the entire group of patients studied, Asp was positively correlated with BMI (p<0.05), M, HGB, total protein, sodium, potassium, and ALAT (p<0.01), and negatively correlated with WBC, chlorides, albumin, and CA15-3 (p<0.05). In the study group, it was positively correlated with M, CA15-3 (p<0.01), HGB, total bilirubin, and potassium, and negatively correlated with NES1 and WBC (p<0.05). In contrast, in the reference group, a positive correlation was found only with sodium (p<0.05), while a negative correlation was found with IgG (p<0.01), ISM1, and PLT (p<0.05).

In patients with G1 malignancy, Asp correlates postitvely with PLT (P<0.05) and negatively with IgM (p<0.05), and in patients with G2 malignancy, it correlates positively with N, chlorides (p<0.01) and vitamin D (p<0.05) and negatively with NES1, M and sodium (p<0.05). In patients with G3 malignancy, Asp correlates positively with IgG (p<0.05) and negatively with sodium (p<0.05).In patients with luminal A cancer, Asp positively correlates with serum creatinine, total bilirubin and IgA (p<0.05) and negatively with CFD, total protein, chlorides, vitamin D (p<0.05). In patients with luminal B HER+ cancer, Asp positively correlates with PLT and ALP (p<0.05) and negatively with vitamin D (p<0.05). In patients with luminal B HER- cancer, it negatively correlates with total bilirubin (p<0.05) and positively with LDH (p<0.01), IgG and ALAT (p<0.05). In patients with TNBC, there was a positive correlation between Asp and CFD (p<0.05) and a negative correlation with LDH (p<0.05).

Considering all patients, CFD positively correlated with PLT (p<0.001), NES1, M, ALP and IgM (p<0.05) and negatively with vitamin D (p<0.01), sodium, ASPAT and ALAT (p<0.05). In the study group, CFD positively correlates with M, PLT and vitamin D (p<0.05) and negatively with HGB, ALP and ASPAT (p<0.05). In the reference group, CFD positively correlates with PLT, ALP, sodium and chlorides (p<0.05) and negatively with IgA and vitamin D (p<0.05). Interestingly, vitamin D deficiency is directly associated with breast cancer (75). In patients with G1 cancer, CFD positively correlates with HGB (p<0.05). In patients with G2 cancer, CFD positively correlates with CA15-3 (p<0.05), a tumor marker for breast cancer, which also explains the highest AUC obtained among the studied adipokines. In this group, it also correlates negatively with T, HGB, CRP and ASPAT (p<0.05). In patients with G3 cancer, CFD negatively correlates with BMI and ALAT (p<0.05). In patients with luminal A cancer a positive correlation was observed for vitamin D (p<0.05), while a negative correlation was observed for Asp and ALAT (p<0.05). In patients with luminal B HER- cancer, a positive correlation with IgG (p<0.05) and a negative correlation with NES1, T, sodium, vitamin D, ALAT (p<0.05). For TNBC patients, only a positive correlation with Asp was observed (p<0.05).

For NES1, in the entire study group, positive correlations were observed with ISM1, WBC (p<0.01), serum creatinine, CRP (p<0.05) and one negative for potassium (p<0.05). For the study group, positive correlations were observed between NES1 and IgA, CRP (p<0.01), HGB, serum creatinine, and potassium (p<0.05), and one negative correlation with Asp (p<0.05). In the reference group, NES1 correlates positively with CA15-3 (p<0.05) and negatively with LDH (p<0.01) and serum creatinine (p<0.05). In the group of patients with G1 malignancy, a positive correlation was observed for ASPAT and ALAT (p<0.05) and a negative correlation with PLT and serum creatinine (p<0.05). For malignancy grade G2, a positive correlation was observed with N (p<0.05) and a negative correlation with IgG (p<0.01), Asp, WBC, ALP, IgM, CRP, CA15-3 (p<0.05) and for G3 a positive correlation with sodium and vitamin D (p<0.05). In patients with luminal A cancer, NES1 positively correlated with total protein, IgM and CRP (p<0.05) and negatively with serum creatinine (p<0.01). In patients with luminal B HER+ cancer, NES1 correlated positively with total protein (p<0.05) and negatively with ISM1 (p<0.05). In patients with luminal B HER-cancer a positive correlation was observed with total protein (p<0.01) and ALP (p<0.05) and a negative correlation with CFD, IgG, and vitamin D (p<0.05).

For ISM1, a positive correlation was observed for NES1, sodium (p<0.01), BMI, PLT, potassium (p<0.05) in the group of all patients and a negative correlation with IgG (p<0.05). In the study group itself, a positive correlation with PLT (p<0.05) and a negative correlation with LDH, sodium, and potassium (p<0.05) were observed. Meanwhile, in the reference group, a positive correlation with CRP and albumin (p<0.05) and a negative correlation with Asp and total bilirubin (p<0.05) were observed. In patients with G2 malignancy, ISM1 positively correlates with chlorides (p<0.01), WBC, IgM and ASPAT (p<0.05) and negatively with ALAT (p<0.01). In patients with G3 malignancy negatively with N (p<0.05). In patients with luminal A cancer, ISM1 positively correlated with WBC (p<0.05), negatively with HGB and LDH (p<0.05). In patients with luminal B HER+ cancer with CA15-3 (p<0.05), and negatively with NES1 (p<0.05). In patients with luminal B HER- cancer, it positively correlated with LDH (p<0.01) and negatively with PGRN (p<0.01) and IgA (p<0.05), and in patients with TNBC, with IgG (p<0.05). Unfortunately, it was not possible to identify a single correlation that would apply to either a single subtype or a single tumor grade. The correlations with vitamin D, BMI, and other adipokines seem interesting, as they may indicate a common direction of biological action.

The study was limited by the small number of patients with specific molecular subtypes. Another clear limitation of the study was the use of serum only, due to the numerous sites of physiological expression of the molecules studied. The analytical limitations of our study should also be taken into account, which is the use of tests designed for scientific research with predefined calibration ranges and sensitivity limits. Statistical analysis of the correlation between the adipokines studied and other parameters proved inconclusive; it was not possible to identify a single parameter that would be specific to a particular type of cancer malignancy or a particular molecular subtype of breast cancer. This indicates a certain distinctiveness and, at the same time, potential pleiotropy of the adipokines studied in the serum of breast cancer patients.

## 5 Conclusions

1. The demonstrated changes in adipsin and isthmin concentrations in breast cancer patients compared to patients with benign tumors may indicate systemic immune and inflammatory response disorders involving the adipokines studied.
2. Interesting observations were also provided by the demonstrated correlations between some of the adipokines and standard laboratory parameters, which may prove helpful in the future in the development of new potential diagnostic and therapeutic algorithms.
3. The demonstrated correlations between the adipokines in the course of breast cancer indicate their multidirectional action in the pathogenesis of breast cancer, which requires further detailed research.

## Data Availability

The original contributions presented in the study are included in the article/**Supplementary Material**. Further inquiries can be directed to the corresponding authors.

## Supplementary material

Supplementary Figure 1 Physiological role of selected adipokines. Created in https://BioRender.com. BAT, brown adipose tissue; HSPC, Hematopoietic stem/progenitor cells.
