# Supplementary material for: Dysregulation of adipokines in the serum of breast cancer patients – role in pathogenesis and potential clinical application (preliminary studies)
Source: Front Immunol. 2026 Mar 31;17:1724900. doi: 10.3389/fimmu.2026.1724900 (PMC13076106; doi:10.3389/fimmu.2026.1724900)
Supplement: Supplementary Figure 1 — Physiological role of selected adipokines. Created in https://BioRender.com. BAT, brown adipose tissue; HSPC, Hematopoietic stem/progenitor cells. [file DataSheet1.docx]

1. **SUPPLEMENTARY MATERIALS**

Table I. Characteristics of selected adipokines

| Adipokine | | Other names | Molecular weight | Structure | Receptors | Physiological expression | Ref. |
| --- | --- | --- | --- | --- | --- | --- | --- |
| Progranulin | | PGRN, acrogranin, proepithelin (PEPI),  GP88, granulin/epithelin precursor (GEP), PC cell-derived growth factor (PCDGF) | 88 kDa | polypeptide chain composed of 593 amino acid residues | EPHA2,  SORT1,  TLR9, TNFR1/2, TNFRSF25 | epithelial cells, endothelium, adipose tissue, skeletal muscle, immune cells, hematopoietic stem cells, neurons | (1–3) |
| FSTL1 | | FSTL1,  transforming growth factor (TFG)-β-induced clone 36 (TSC-36), Follistatin-related protein (FRP) | 46 kDa | 308 [aminoacids](https://www.sciencedirect.com/topics/biochemistry-genetics-and-molecular-biology/amino-acids) in length with five distinct domains: a follistatin-like domain, a Kazal-like domain, two extracellular EF-hand calcium-binding domains, and a Von Willebrand factor-type C domain | BMP,  TLR4, CD14, DIP2A | mammalian tissues and cells of mesenchymal origin | (4–10) |
| Asprosin | Asp | ∼30 kDa | profibrillin C-terminal polypeptide | OR4M1 | white adipose tissue | (11–14) |  |
| Meteorin | METRN | ∼30 kDa | protein of 293 amino acids | not yet characterized | central and peripheral nervous system, bone marrow macrophages, in mouse -  brain, kidney, heart, ovary and skeletal muscle | (15–19) |  |
| Adipsin | Complement factor D (CFD) | 24 kDa | serine protease comprising 228 amino acids | C3aR | adipocytes | (20–22) |  |
| Nesfatin | Nucleobindin-2 (NUCB2) | 9.8 kDa | 82 amino acids peptide | GPCRs 3, 6 & 12 | stomach, pancreas, heart, reproductive organs, and adipose tissue | (23–28) |  |
| Neuregulin 4 | NRG4 | ~6.7 KDa | growth factor related to epidermal growth factor | ErbB4 | brown adipose tissue, nervous system, muscles, pancreas | (29–32) |  |
| Isthmin | ISM1 | ~60 kDa | protein with 499 amino acids that contains 3 α-helices and 2 β-sheets | Integrin αvβ5  GRP78 | human tissues of lung, liver, breast, brain, stomach, muscle, skin, bone marrow, and colon | (33–35) |  |

1. Abbreviations: DIP2A - Disco Interacting Protein 2 Homolog A, EPHA2 - ephrin type-A receptor 2,  ErbB4 - erb-b2 receptor tyrosine kinase 4, GPCRs - G protein-coupled receptors, GRP78 - glucose-regulated protein, SORT1 – sortilin, TLR4 - Toll-like receptor-four, TLR9 - Toll-like receptor 9, TNFR1/2 - tumor necrosis factor receptor 1/2, TNFRSF25 - tumor necrosis factor receptor superfamily member 25

The physiological role of the adipokines studied is presented in Figure 1 (1,7,14,20,21,24,29,33,33,34,36–41).
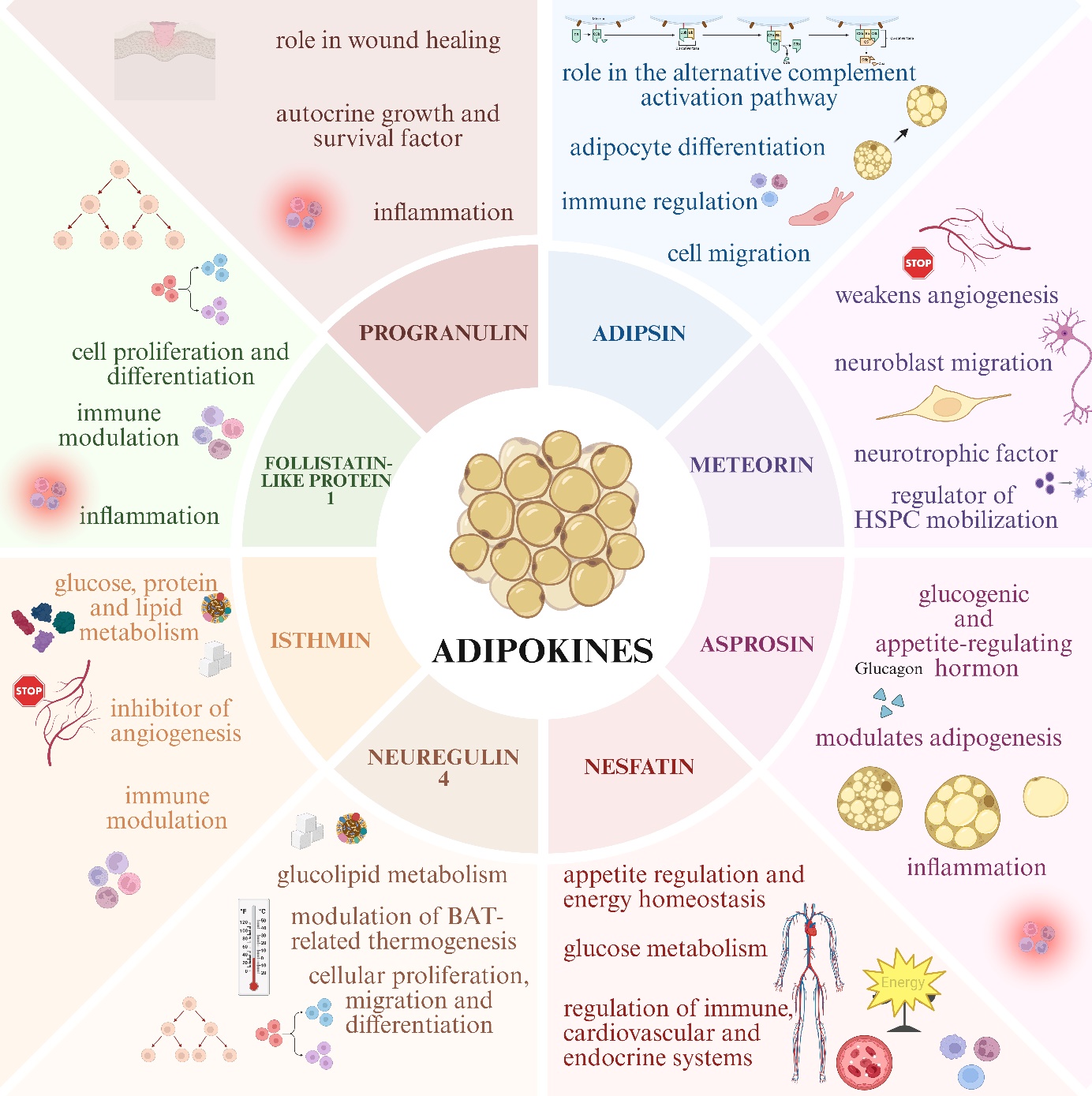


Created in [https://BioRender.com](https://biorender.com/)

Abbreviations: BAT - brown adipose tissue, HSPC - Hematopoietic stem/progenitor cells

Fig. 1. Physiological role of selected adipokines.

1. Purrahman D, Mahmoudian-Sani M-R, Saki N, Wojdasiewicz P, Kurkowska-Jastrzębska I, Poniatowski ŁA. Involvement of progranulin (PGRN) in the pathogenesis and prognosis of breast cancer. *Cytokine* (2022) 151:155803. doi: 10.1016/j.cyto.2022.155803

2. Berger K, Persson E, Gregersson P, Ruiz-Martínez S, Jonasson E, Ståhlberg A, Rhost S, Landberg G. Interleukin-6 Induces Stem Cell Propagation through Liaison with the Sortilin-Progranulin Axis in Breast Cancer. *Cancers (Basel)* (2023) 15:5757. doi: 10.3390/cancers15245757

3. Olczak M, Poniatowski ŁA, Siwińska A, Kwiatkowska M, Chutorański D, Wierzba-Bobrowicz T. Elevated serum and urine levels of progranulin (PGRN) as a predictorof microglia activation in the early phase of traumatic brain injury:a further link with the development of neurodegenerative diseases. *fn* (2021) 59:81–90. doi: 10.5114/fn.2021.105137

4. Yang Y, Lu T, Jia X, Gao Y. FSTL1 Suppresses Triple-Negative Breast Cancer Lung Metastasis by Inhibiting M2-like Tumor-Associated Macrophage Recruitment toward the Lungs. *Diagnostics (Basel)* (2023) 13:1724. doi: 10.3390/diagnostics13101724

5. Zhang Y, Xu X, Yang Y, Ma J, Wang L, Meng X, Chen B, Qin L, Lu T, Gao Y. Deficiency of Follistatin-Like Protein 1 Accelerates the Growth of Breast Cancer Cells at Lung Metastatic Sites. *J Breast Cancer* (2018) 21:267–276. doi: 10.4048/jbc.2018.21.e43

6. Yang Y, Mu T, Li T, Xie S, Zhou J, Liu M, Li D. Effects of FSTL1 on the proliferation and motility of breast cancer cells and vascular endothelial cells. *Thorac Cancer* (2017) 8:606–612. doi: 10.1111/1759-7714.12491

7. Du R, Li K, Guo K, Chen Z, Han L, Bian H. FSTL1: A double-edged sword in cancer development. *Gene* (2024) 906:148263. doi: 10.1016/j.gene.2024.148263

8. Wang Y, Li D, Xu N, Tao W, Zhu R, Sun R, Fan W, Zhang P, Dong T, Yu L. Follistatin-like protein 1: a serum biochemical marker reflecting the severity of joint damage in patients with osteoarthritis. *Arthritis Res Ther* (2011) 13:R193. doi: 10.1186/ar3522

9. Maksimowski NA, Song X, Bae EH, Reich H, John R, Pei Y, Scholey JW, Nephrotic Syndrome Study Network Neptune null. Follistatin-Like-1 (FSTL1) Is a Fibroblast-Derived Growth Factor That Contributes to Progression of Chronic Kidney Disease. *Int J Mol Sci* (2021) 22:9513. doi: 10.3390/ijms22179513

10. Mattiotti A, Prakash S, Barnett P, van den Hoff MJB. Follistatin-like 1 in development and human diseases. *Cell Mol Life Sci* (2018) 75:2339–2354. doi: 10.1007/s00018-018-2805-0

11. Akkus G, Koyuturk LC, Yilmaz M, Hancer S, Ozercan IH, Kuloglu T. Asprosin and meteorin-like protein immunoreactivity in invasive ductal breast carcinoma stages. *Tissue and Cell* (2022) 77:101855. doi: 10.1016/j.tice.2022.101855

12. Kocaman N, Onat E, Balta H, Üçer Ö. Are Meteorin-Like Peptide and Asprosin Important in the Diagnosis of Breast Tumors? *Cureus* (2024) 16:e62979. doi: 10.7759/cureus.62979

13. Ovali MA, Bozgeyik I. Asprosin, a C-Terminal Cleavage Product of Fibrillin 1 Encoded by the ***FBN1*** Gene, in Health and Disease. *Mol Syndromol* (2022) 13:175–183. doi: 10.1159/000520333

14. Romere C, Duerrschmid C, Bournat J, Constable P, Jain M, Xia F, Saha PK, Del Solar M, Zhu B, York B, et al. Asprosin, a Fasting-Induced Glucogenic Protein Hormone. *Cell* (2016) 165:566–579. doi: 10.1016/j.cell.2016.02.063

15. Sankaranarayanan I, Tavares-Ferreira D, He L, Kume M, Mwirigi JM, Madsen TM, Petersen KA, Munro G, Price TJ. Meteorin Alleviates Paclitaxel-Induced Peripheral Neuropathic Pain in Mice. *J Pain* (2023) 24:555–567. doi: 10.1016/j.jpain.2022.10.015

16. Jørgensen JR, Thompson L, Fjord-Larsen L, Krabbe C, Torp M, Kalkkinen N, Hansen C, Wahlberg L. Characterization of Meteorin—An Evolutionary Conserved Neurotrophic Factor. *J Mol Neurosci* (2009) 39:104–116. doi: 10.1007/s12031-009-9189-4

17. Wang L, Huang G, Xiao H, Leng X. A pan-cancer analysis of the association of METRN with prognosis and immune infiltration in human tumors. *Heliyon* (2024) 10:e37213. doi: 10.1016/j.heliyon.2024.e37213

18. Nishino J, Yamashita K, Hashiguchi H, Fujii H, Shimazaki T, Hamada H. Meteorin: a secreted protein that regulates glial cell differentiation and promotes axonal extension. *EMBO J* (2004) 23:1998–2008. doi: 10.1038/sj.emboj.7600202

19. Delaunay K, Sellam A, Dinet V, Moulin A, Zhao M, Gelizé E, Canonica J, Naud M-C, Crisanti-Lassiaz P, Behar-Cohen F. Meteorin Is a Novel Therapeutic Target for Wet Age-Related Macular Degeneration. *JCM* (2021) 10:2973. doi: 10.3390/jcm10132973

20. Taylor EB. The complex role of adipokines in obesity, inflammation, and autoimmunity. *Clin Sci (Lond)* (2021) 135:731–752. doi: 10.1042/CS20200895

21. Khaledian B, Thibes L, Shimono Y. Adipocyte regulation of cancer stem cells. *Cancer Science* (2023) 114:4134–4144. doi: 10.1111/cas.15940

22. Barratt J, Weitz I. Complement Factor D as a Strategic Target for Regulating the Alternative Complement Pathway. *Front Immunol* (2021) 12:712572. doi: 10.3389/fimmu.2021.712572

23. Ning S, Liu C, Wang K, Cai Y, Ning Z, Li M, Zeng L. Correction: NUCB2/Nesfatin-1 drives breast cancer metastasis through the up-regulation of cholesterol synthesis via the mTORC1 pathway. *J Transl Med* (2023) 21:524. doi: 10.1186/s12967-023-04385-z

24. Kmiecik AM, Dzięgiel P, Podhorska-Okołów M. Nucleobindin-2/Nesfatin-1-A New Cancer Related Molecule? *Int J Mol Sci* (2021) 22:8313. doi: 10.3390/ijms22158313

25. Kmiecik A, Ratajczak-Wielgomas K, Grzegrzółka J, Romanowicz H, Smolarz B, Dziegiel P. Expression of NUCB2/NESF-1 in Breast Cancer Cells. *Int J Mol Sci* (2022) 23:9177. doi: 10.3390/ijms23169177

26. Shin E, Koo JS. The Role of Adipokines and Bone Marrow Adipocytes in Breast Cancer Bone Metastasis. *Int J Mol Sci* (2020) 21:4967. doi: 10.3390/ijms21144967

27. Zhou S, Nao J. Nesfatin-1: A Biomarker and Potential Therapeutic Target in Neurological Disorders. *Neurochem Res* (2024) 49:38–51. doi: 10.1007/s11064-023-04037-0

28. Aydin S. Role of NUCB2/nesfatin-1 as a Possible Biomarker. *CPD* (2013) 19:6986–6992. doi: 10.2174/138161281939131127143422

29. Liu Y, Chen M. Neuregulin 4 as a novel adipokine in energy metabolism. *Front Physiol* (2022) 13:1106380. doi: 10.3389/fphys.2022.1106380

30. Dan X, Li K, Xu J, Yan P. The Potential of Neuregulin 4 as a Novel Biomarker and Therapeutic Agent for Vascular Complications in Type 2 Diabetes Mellitus. *JIR* (2024) Volume 17:8543–8554. doi: 10.2147/JIR.S492115

31. Howard L, Wyatt S, Davies AM. Neuregulin‐4 contributes to the establishment of cutaneous sensory innervation. *Developmental Neurobiology* (2021) 81:139–148. doi: 10.1002/dneu.22803

32. Hayes NVL, Blackburn E, Smart LV, Boyle MM, Russell GA, Frost TM, Morgan BJT, Baines AJ, Gullick WJ. Identification and Characterization of Novel Spliced Variants of Neuregulin 4 in Prostate Cancer. *Clinical Cancer Research* (2007) 13:3147–3155. doi: 10.1158/1078-0432.CCR-06-2237

33. Turk A, Metin TO, Kuloglu T, Yilmaz M, Artas G, Ozercan IH, Hancer S. Isthmin-1 and spexin as promising novel biomarker candidates for invasive ductal breast carcinoma. *Tissue and Cell* (2024) 91:102601. doi: 10.1016/j.tice.2024.102601

34. Shakhawat HM, Hazrat Z, Zhou Z. Isthmin-A Multifaceted Protein Family. *Cells* (2022) 12:17. doi: 10.3390/cells12010017

35. Hu M, Zhang X, Hu C, Teng T, Tang Q-Z. A brief overview about the adipokine: Isthmin-1. *Front Cardiovasc Med* (2022) 9:939757. doi: 10.3389/fcvm.2022.939757

36. Sat-Muñoz D, Martínez-Herrera B-E, Quiroga-Morales L-A, Trujillo-Hernández B, González-Rodríguez J-A, Gutiérrez-Rodríguez L-X, Leal-Cortés C-A, Portilla-de-Buen E, Rubio-Jurado B, Salazar-Páramo M, et al. Adipocytokines and Insulin Resistance: Their Role as Benign Breast Disease and Breast Cancer Risk Factors in a High-Prevalence Overweight-Obesity Group of Women over 40 Years Old. *Int J Environ Res Public Health* (2022) 19:6093. doi: 10.3390/ijerph19106093

37. Serrero G. Potential of Theranostic Target Mining in the Development of Novel Diagnostic and Therapeutic Products in Oncology: Progranulin/GP88 as a Therapeutic and Diagnostic Target for Breast and Lung Cancers. *Rinsho Byori* (2016) 64:1296–1309.

38. Dai Y-W, Ma J-K, Jiang R, Zhan X-L, Chen S-Y, Feng L-L, Zhang Q, Liang T-B, Lv K, Yang G-J, et al. Meteorin links the bone marrow hypoxic state to hematopoietic stem/progenitor cell mobilization. *Cell Reports* (2022) 40:111361. doi: 10.1016/j.celrep.2022.111361

39. Liu C, Kong N, Liu H, Zhang Y, Qin W, Zhao W, Yang X, Wang Y, Cao X, Liu T, et al. FSTL1 and TLR4 interact with PEDV structural proteins to promote virus adsorption to host cells. *J Virol* (2025) 99:e0183724. doi: 10.1128/jvi.01837-24

40. Wang H, Wang L, Hu F, Wang P, Xie Y, Li F, Guo B. Neuregulin-4 attenuates diabetic cardiomyopathy by regulating autophagy via the AMPK/mTOR signalling pathway. *Cardiovasc Diabetol* (2022) 21:205. doi: 10.1186/s12933-022-01643-0

41. Khan S, Syeda S, Raghuvanshi Y, Shrivastava A. Pathophysiological insights into asprosin: an emerging adipokine in reproductive health. *Rev Endocr Metab Disord* (2025) doi: 10.1007/s11154-025-09975-4
